# Exercise with fasting or isotonic drink? A randomized controlled trial in youth elite basketball players

**DOI:** 10.1080/15502783.2025.2528533

**Published:** 2025-07-08

**Authors:** Petra Márton, Luca Kata Bátai, Titanilla Takács, Emese Csulak, Anna Réka Kiss, Bence Kopper, Liliána Erzsébet Szabó, Dorottya Balla, Iván Petrov, Lilla Lázár, Hajnalka Vágó, Béla Merkely, Nóra Sydó

**Affiliations:** a Semmelweis University, Heart and Vascular Centre, Budapest, Hungary; b Semmelweis University, Department of Sports Medicine, Budapest, Hungary; c Győr-Moson-Sopron County Petz Aladár University Teaching Hospital, Department of Cardiology, Győr, Hungary; d University of Physical Education, Department of Kinesiology, Budapest, Hungary

**Keywords:** Sports performance, nutrition, isotonic drink, carbohydrate, basketball

## Abstract

**Background:**

Pre-exercise carbohydrate intake is known to influence performance; however, data describing their effect on cardiorespiratory parameters is scarce. This study aimed to assess the effects of isotonic drink consumption on cardiopulmonary exercise test (CPET) parameters in elitemale youth basketball players.

**Methods:**

The athletes were randomized into a fasting (400 ml mineral water) and an isotonic drink (400 ml 7% isotonic solution) group respectively, and consumed the drinks 30 minutes before the CPET. Pre-, peak- and post-CPET glucose levels were measured. Borg and lactate were assessed every 2 minutes during the test.

**Results:**

Seventy-one athletes (age: 15.9 ± 1.8 years) were included in the study. The isotonic drink group had higher pre- and post-CPET glucose levels (*p* < 0.05). They reported a lower Borg scale at the 2nd, 6th, and 10th minutes (*p* < 0.05), while their lactate levels were lower at the 14th minute (*p* < 0.05). Regression analysis showed that fasting was associated with higher Borg scale ratings (β-coefficient: 0.72, *p* < 0.001) and increased lactate accumulation over time (β-coefficient: 0.13, *p* = 0.01). No difference was found in exercise duration or maximal aerobic capacity.

**Conclusions:**

Single-dose isotonic drink consumption before CPET reduces perceived exertion and moderates lactate accumulation, which may suggest a beneficial effect during the exercise test.

## 1. Introduction

Optimizing performance and recovery through nutritional interventions is crucial for young athletes [1,2]. Carbohydrate intake has been extensively studied due to its potential benefits on endurance, energy levels, and overall athletic performance [3–5]. Isotonic drinks are designed to quickly replenish fluids, electrolytes, and energy, which helps maintain blood glucose levels, delay fatigue, and sustain performance [6–8]. However, clinical evidence is limited regarding the optimal quantity, composition, and timing of isotonic drink consumption in youth athletes [9]. These factors may vary according to age, sex, anthropometric characteristics, type of sport, and training phases [10]. The most accurate measurement of performance and cardiorespiratory fitness is cardiopulmonary exercise testing (CPET). This can assess not only the cardiorespiratory and pulmonary function but also the metabolic responses of the athletes [11]. Lactate measurements during CPET provide information about the metabolic status of the athletes. Pre-exercise carbohydrate availability may also influence lactate accumulation, since muscle glycogen content is a key determinant of glycolytic flux and lactate kinetics during maximal exercise [12]. While lactate is a well-known marker of anaerobic metabolism, fatigue during high-intensity exercise is primarily driven by various factors, including proton and inorganic phosphate accumulation, and ionic imbalances [13–15].

Although isotonic drinks are typically recommended for prolonged endurance events to maintain carbohydrate availability and fluid balance, their potential benefits in relatively short, high-intensity exercise remain unclear. In athletes, maximal CPET requires maximal exertion and relies heavily on anaerobic metabolism, where pre-exercise glycogen stores and blood glucose levels are potentially relevant. However, the current guidelines recommend that CPET should be performed after 2 hours of fasting [11], but this method may not reflect the everyday practice and performance of the athletes [7]. Modifying the laboratory testing protocol or using simulated plays may help assess the peak performance of competitive athletes more accurately and address concerns regarding ecological validity [16].

In this study, we aim to evaluate the effect of isotonic drinks during CPET in a randomized controlled trial of elite [17] male youth basketball players. We sought to determine whether consuming isotonic drinks or just mineral water before an exercise test may lead to improvement in key performance parameters. Our second aim was to evaluate the differences in CPET results in fasting and non-fasting environments.

## 2. Materials and methods

### 2.1. Study design

This study employed a randomized controlled trial design to evaluate the effects of fasting versus isotonic drink consumption on athletic performance. The study population comprised an entire basketball academy, consisting of male youth athletes with a uniform training schedule and dietary supervision by a registered sports dietitian. All athletes underwent comprehensive sports cardiology screening, including a dietary questionnaire, laboratory tests, resting 12-lead electrocardiogram (ECG), echocardiography, and CPET with fingertip glucose and lactate measurements.

Participants were divided equally into two groups: a fasting group and an isotonic drink group. We used single-blind, stratified randomization. First, athletes were stratified by age group. Then, using simple randomization via a computer-generated randomization schedule, participants were allocated to one of the two groups in a 1:1 ratio, referring to a table of random numbers. Randomization was performed by a dedicated member of the research team who was not involved in the cardiology screening or the administration of the CPET.

Only the dietitian who prepared and distributed the drinks was unblinded; the physicians conducting the CPET were blinded to group assignments until the conclusion of the study. All drinks—400 ml of either mineral water or a transparent, uncolored 7% isotonic solution (Gatorade®: 28 g carbohydrates, 182 mg sodium, 60.9 mg potassium) – were served in nontransparent bottles 30 minutes before the CPET. The volume of 400 ml was selected based on current sports nutrition guidelines recommending 5–7 ml/kg of fluid intake prior to high-intensity exercise [18,19], which aligned with 4.7–6.6 ml/kg in our cohort based on body mass.

**Figure 1.**
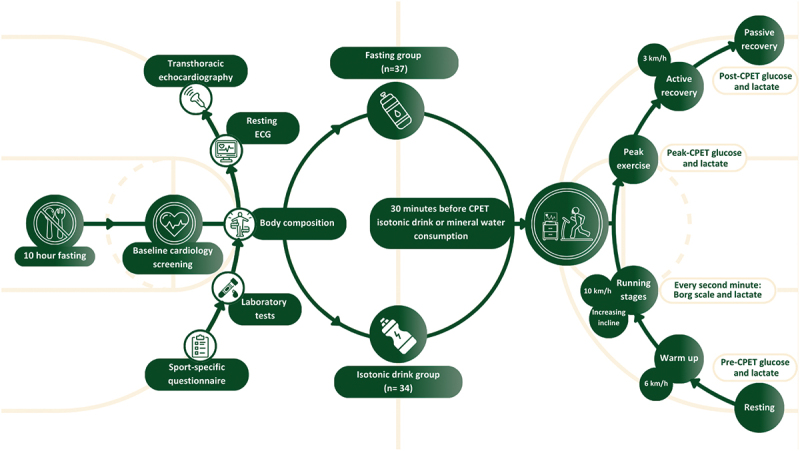
Study design and interventions.

Importantly, all athletes had previously undergone CPET and provided consent for the examination. However, they were not informed of the research objective, and thus were unaware that subjective ratings such as the Borg scale were of particular interest or that differences across groups were expected. While athletes may have recognized whether they received water or an isotonic drink, they had no knowledge of the study’s hypothesis or anticipated outcomes. This helped reduce expectation bias and supports the validity of differences observed in subjective measures. Study design and interventions are illustrated in Figure 1.

CPET=cardiopulmonary exercise testing, ECG=electrocardiogram

### 2.2. Assessments

Initially, the athletes completed a comprehensive sport-specific questionnaire. This questionnaire collected detailed information on their personal and family history, cardiovascular risk factors, and current use of medication or dietary supplements. The study ensured consistent dietary intake by providing the participants with their primary meals at the Academy. Additionally, a thorough nutritional evaluation was conducted using a specialized dietary assessment that included a food frequency questionnaire designed for athletes.

Laboratory tests were conducted for all the athletes, including qualitative and quantitative blood counts, assessment of renal and liver function, ion concentrations, C-reactive protein level, iron panel (serum iron, ferritin, transferrin, transferrin saturation, soluble transferrin receptor), thyroid hormones (thyroid stimulating hormone, T3, T4), folic acid, vitamin B12, and vitamin D levels.

Body composition was analyzed using the InBody 770 (InBody Co. Ltd., Seoul, Korea) to measure weight, body fat mass, and fat-free mass, including skeletal muscle mass, body cell mass, total body water, and bone mineral mass. Height was measured with a stadiometer (InBody BSM370, InBody Co. Ltd., Seoul, Korea).

A standard resting 12-lead ECG (CardioSoft PC, General Electric Healthcare, Helsinki, Finland) was recorded, and blood pressure was measured using the Omron M6 Comfort (HEM-7360-E) machine. Transthoracic echocardiography provided comprehensive information about cardiac structure and function. The measurements followed the latest guidelines [20,21].

### 2.3. CPET protocol

The CPET examinations were conducted on a treadmill ergometer using a protocol designed for dryland athletes. The protocol and external load standardization were ensured by recruiting participants from the same basketball academy with identical training schedules, supervised dietary intake, and comparable training phases. The participants had an approximate weekly MET load of 111.2. This controlled environment allowed for reproducibility and comparability of exercise responses across participants [22]. It started with a 1-minute warm-up at 6 km/h, followed by running at a constant speed of 10 km/h with an inclination of 1% every minute. Athletes were not allowed to hold the handrail. The active recovery lasted for one minute walking at 3 km/h, followed by four minutes of passive recovery in a sitting position. During the test, ECG was continuously monitored, and blood pressure was measured every three minutes. The following cardiorespiratory parameters were measured: load time, maximum heart rate (HR), HR recovery (calculated as the difference between peak HR and HR measured after 1 minute of active recovery), absolute maximum aerobic capacity (VO_2_ max), relative maximum aerobic capacity (VO2 max), minute ventilation (VE), ventilation/volume of exhaled carbon dioxide (VE/VCO2), and respiratory exchange ratio (RER). The ratings of perceived exertion (Borg scale, 6–20 [23]) and capillary lactate concentrations were measured simultaneously every two minutes during the CPET (Lactate Scout EKF Diagnostics, Germany). Alongside with the resting (pre-CPET), peak (peak-CPET), and 5-minute recovery (post-CPET) lactate measurements blood glucose levels (Dcont IDEÁL device, 77 Elektronika Kft, Hungary) were also obtained from the fingertip. In the isotonic drink group, resting glucose levels were measured twice: upon arrival and after consuming the isotonic drink (pre-CPET). In the fasting group, the resting glucose levels were measured only once during the pre-CPET measurements, as no carbohydrate intake occurred before the CPET. The test was considered maximal if the RER exceeded 1.1, or if the participant achieved the predicted peak oxygen uptake or a plateau, additionally if they reached their predicted maximal work rate or heart rate, showed ventilatory limitation, or reported exhaustion with a Borg scale rating.

### 2.4. Statistical analysis

The statistical analysis and figure generation were performed using R version 4.2.2. and R-studio. Normal distribution was assessed with Q-Q plots and the Shapiro-Wilk test. Continuous parameters are described as mean and standard deviation (SD). A pairwise comparison of continuous variables was performed using a Student’s t-test. The correlation between continuous metrics was calculated using Pearson’s tests. Linear regression models were used to assess the association between study groups (isotonic drink vs. fasting) and cardiopulmonary exercise test (CPET) outcomes. For parameters recorded at a single time point (e.g. VO₂ max, heart rate recovery), separate linear regression models were fitted for each variable. For time-dependent outcomes (lactate levels and Borg scale ratings), we used linear models including an interaction term between time and fluid consumption (Time × Group) to assess differential trends over the course of the exercise test. Because lactate values were positively skewed, they were log-transformed prior to modeling to meet assumptions of normality. Model assumptions were evaluated using standard residual diagnostics, including checks for linearity, normality of residuals, homoscedasticity, and the presence of influential data points (see Supplementary Figures S2–3). All assumptions were found to be adequately met. Effect estimates are reported as unstandardized regression coefficients (B) with corresponding 95% confidence intervals. For multiple comparisons involving CPET parameters, false discovery rate (FDR) correction was applied. A p-value of < 0.05 was considered statistically significant.

Based on our expert clinical experience, a 10% difference in VO₂ max was considered physiologically meaningful in highly trained populations. Given that our cohort had expected VO₂ max values above 50 mL·kg^− 1^ ·min^− 1^, we defined a relevant group difference as 5 mL·kg^− 1^ ·min^− 1^. Conservatively assuming a standard deviation of 5.0, this corresponds to an effect size of Cohen’s *d* = 1.0. Our a priori sample size calculations indicated that 21 participants per group would be sufficient to detect such a difference with 90% power at α = 0.05. With our actual sample size of 35 per group and an observed standard deviation of 4.0, post hoc power to detect a 5 mL·kg^− 1^ ·min^− 1^ difference was approximately 99.8%.

## 3. Results

### 3.1. Study population

In total, we enrolled 71 elite male basketball players (age: 15.9 ± 1.8 years, training hours: 17.1 ± 4.3 hours) from a basketball academy. There was no difference in the baseline characteristics between the fasting and the isotonic groups (Table 1).

**Table 1.** Baseline characteristics of the fasting and of the isotonic drink groups.

	Total (*n*= 71)	Fasting Group (*n*= 37)	Isotonic Group (*n*=34)	P
Age (years)	15.9 ± 1.8	15.9 ± 1.5	16.2 ± 2.1	0.480
Sports experience (years)	8.5 ± 3.4	8.7 ± 3.8	8.2 ± 3.0	0.522
Training hours/week	17.1 ± 4.3	17.6 ± 4.4	16.6 ± 4.2	0.317
Body height (cm)	186.7 ± 9.0	186.3 ± 10.6	187.0 ± 6.0	0.755
Skeletal muscle mass (kg)	38.3 ± 5.2	38.5 ± 6.2	38.0 ± 4.2	0.689
Body fat percentage (%)	9.0 ± 2.9	9.3 ± 2.1	8.5 ± 2.5	0.145
Total body mass (kg)	73.5 ± 12.5	73.9 ± 14.6	72.5 ± 7.6	0.640

Table legend: Pairwise comparison between the fasting and the isotonic groups. Continuous variables are shown as mean ± SD.

Regarding the sports‐specific questionnaire, no athlete reported any medical complaint, while positive family history was revealed in 3 (4.2%) cases. During the personal dietary consultation, food intolerances were rare: gluten sensitivity (n = 1), lactose intolerance and gluten sensitivity (n = 1), and restraint from pork consumption due to religious reasons (n = 1). There was no vegetarian among the players. According to the food frequency questionnaire, the primary carbohydrate sources among the study participants included fresh and dried fruits, potato, whole grain bread, refined wheat flour bread, muesli or cornflakes, oatmeal, cakes, sweets, and cocoa products (Table 2). All the athletes followed a balanced diet, rich in essential nutrients. Overall, the two groups reported similar food intake frequencies, which suggests no major difference in glycogen storage load. Regarding fluid intake during training, 98% of the athletes consumed isotonic drinks. 70% of the athletes consumed 1.5–2 liters of fluids per average training session, while 30% consumed 0.5–1 liter.

**Table 2.** Consumption of the main carbohydrate sources of the fasting and of the isotonic drink groups according to the food frequency questionnaire.

Food group	Number of consumptions Fasting Group (n=37)			Number of consumptions Isotonic Group (n=34)		
	1–2x/day	3–4x/day	5–6x/day	1–2x/day	3–4x/day	5–6x/day
Fruit – fresh, raw	10 (27%)	10 (27%)	0	14 (42%)	11 (32%)	0
Fruits - dried fruit	3 (8%)	0	0	1 (3%)	0	0
Potato	8 (21%)	1 (3%)	1 (3%)	6 (18%)	1 (3%)	0
Whole grain, brown bread	6 (16%)	2 (5%)	2 (5%)	5 (15%)	5 (15%)	0
Refined wheat flour bread	10 (27%)	23 (62%)	0	8 (24%)	20 (59%)	3 (9%)
Muesli, cornflakes	7 (19%)	3 (8%)	0	7 (21%)	4 (12%)	0
Oats, oatmeal	15 (40%)	5 (13%)	0	10 (29%)	13 (38%)	0
Cake, sweets	10 (27%)	2 (5%)	0	10 (29%)	10 (29%)	0
Cocoa products (70%), chocolate	4 (11%)	2 (5%)	0	3 (9%)	0	0

In the laboratory exams, no major alteration was found. Low iron storage [24] was revealed in 25 cases, and low vitamin D levels in 40 cases, with no difference between the groups. All athletes had normal laboratory fasting blood glucose values.

Due to high resting or exercise blood pressure, 24‐hour ambulatory blood pressure monitoring was performed in 4 cases (6%). Hypertension was diagnosed in one case, consequently an angiotensin receptor blocker therapy was initiated. All resting ECG and echocardiography parameters were in the athletic physiologic range [25–27].

On the CPET exams, the athletes showed good cardiorespiratory fitness (Isotonic vs. Fasting group relative VO_2_ max: 56.7 ± 4.1 vs. 55.6 ± 3.1 mL·kg-1·min-1 *p* = 0.189), and there was no difference between the CPET parameters of the two groups (Table 3). The association between CPET performance outcomes and group assignment (Isotonic vs. Fasting) is reported in detail in Supplementary Table S1, along with normality diagnostics in Supplementary Figure S1 (Q-Q plots). No arrhythmia or other pathologic alteration was revealed.

**Table 3.** Cardiopulmonary exercise test parameters and blood glucose levels in the fasting and in the isotonic drink groups.

	Total (*n*= 71)	Fasting Group (*n*= 37)	Isotonic Group (*n*= 34)	P
Load Time (min)	12.8 ± 1.6	12.9 ± 1.7	12.7 ± 1.4	0.489
Maximum HR (bpm)	195 ± 8	196 ± 9	195 ± 7	0.599
HR recovery (bpm)	29 ± 8	32 ± 5	29 ± 6	0.373
Absolute VO_2_ max (L/min)	4.1 ± 0.7	4.1 ± 0.5	4.2 ± 0.8	0.829
Relative VO_2_ max (mL·kg-1·min-1)	56.3 ± 3.9	55.6 ± 3.1	56.7 ± 4.1	0.189
Minute ventilation (L/min)	149.0 ± 21.4	147.9 ± 27.1	149.5 ± 13.1	0.765
VE/VCO_2_	33.7 ± 4.0	33.0 ± 3.4	33.9 ± 3.9	0.321
RER	1.1 ± 0.1	1.1 ± 0.1	1.1 ± 0.1	0.797
Pre-CPET lactate (mmol/L)	1.0 ± 0.4	1.0 ± 0.4	0.9 ± 0.3	0.580
Peak-CPET lactate (mmol/L)	8.9 ± 2.2	9.0 ± 2.0	8.7 ± 2.0	0.439
Post-CPET lactate (mmol/L)	9.2 ± 2.5	9.2 ± 2.4	9.1 ± 2.1	0.892
Pre-CPET glucose (mmol/L)	6.4 ± 1.4	5.5 ± 0.5	7.7 ± 1.1	<.001*
Peak-CPET glucose (mmol/L)	5.7 ± 0.9	5.6 ± 1.1	5.9 ± 0.7	0.168
Post-CPET glucose (mmol/L)	7.1 ± 1.3	6.6 ± 1.0	7.3 ± 1.0	0.001*

Table legend: Pairwise comparisons between the fasting and the isotonic groups. Continuous variables are shown as mean ± SD. Abbreviations: HR = heart rate, bpm = beats per minute, CPET = Cardiopulmonary exercise test, RER = Respiratory exchange ratio, SD = Standard deviation, VCO_2_ = volume of exhaled carbon dioxide, VE = minute ventilation, VE/VCO_2_ = ventilation/volume of exhaled carbon dioxide, VO_2_ max = maximum aerobic capacity.

The fasting blood glucose levels (measured upon arrival) were similar in the two groups (Isotonic vs. Fasting group: 5.5 ± 0.5 vs. 5.3 ± 0.5 mmol/L, p = 0.305). Pre-CPET and post-CPET glucose levels were higher in the isotonic group (p < 0.001).

### 3.2. Association between isotonic drink consumption and exertion levels

**Figure 2.**
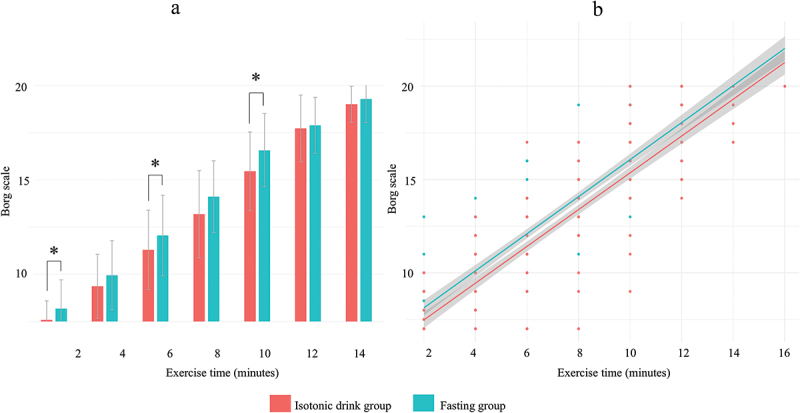
Longitudinal tracking of the Borg scale during CPET exam in the fasting and in the isotonic drink groups.

During the CPET exam, the 2-minute Borg values increased with exercise. There was a trend indicating that the fasting group showed higher values in Borg. On pairwise comparisons, the isotonic drink group rated their exertion levels significantly lower than the fasting group in the 2nd minute (Isotonic vs. Fasting group: 7.3 ± 0.6 vs. 8.0 ± 1.2, *p* = 0.03), 6th min (11.1 ± 1.9 vs. 12.2 ± 2.0, *p* = 0.024) and 10th min (15.5 ± 2.1 vs. 16.6 ± 1.9, *p* = 0.023) of the exercise time (Figure 2).

Overall trend of Borg Scale shown on bar chart (A) and scatter plot (B) in the Isotonic drink (red) and in the fasting group (green). A: Pairwise comparisons between the fasting and isotonic groups. Continuous variables are shown as mean±SD. B: Borg scale ratings plotted over time with fitted regression lines for each group. The shaded areas represent the 95% confidence intervals around the regression lines. * indicates statistical significance (*p*<0.05).

Moreover, we found a significant positive association between the Borg scale ratings and fasting state (unstandardized B coefficient: 0.72, 95% CI [0.35, 1.08], *p* < 0.001) adjusted for load time (Figure 2, Model diagnostics are shown in Supplementary Figure S2). The effect of fasting on Borg ratings remained consistent over time, showing no significant interaction (unstandardized B-coefficient: 0.00, 95% CI [−0.11, 0.1], *p* = 0.93).

### 3.3. Association between isotonic drink consumption and lactate levels

Lactate levels were also tracked throughout the examination: pre-CPET, every 2 minutes during the exercise test, and post-CPET. On pairwise comparison from the 6th minute onwards, lactate levels were consistently but non-significantly lower in the isotonic drink group than the fasting group. While lactate levels differed significantly between groups at the 14-minute mark (Isotonic: 7.2 ± 3.3 vs. Fasting: 9.7 ± 2.1 mmol/L, *p* = 0.037), this comparison reflects only the subset of participants who completed the test up to this time point (27% of the sample). Peak-CPET lactate levels showed no significant difference between the groups.

**Figure 3.**
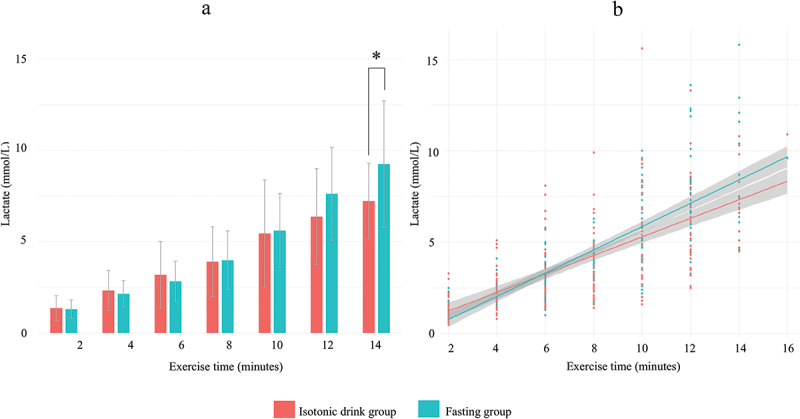
Longitudinal tracking of lactate levels during the CPET exam in the fasting and in the isotonic drink groups.

In the log-transformed linear regression model, lactate levels increased significantly with load time (B = 0.15, 95% CI [0.13, 0.16], *p* < 0.001). There was no significant overall difference in lactate levels between the fasting and isotonic groups (*p* = 0.22). However, we observed a significant positive interaction between load time and fasting status (B = 0.023, 95% CI [0.001, 0.044], *p* = 0.041). This indicates that the rate of lactate accumulation over time was modestly but significantly higher in the fasting group compared to the isotonic group (Figure 3, Model diagnostics: Supplementary Figure S3).

Overall trend of lactate levels shown on bar chart (a) and scatter plot (b) for the Isotonic drink group (red) and the Fasting group (green). A: Pairwise comparisons between groups at each time point; values are presented as mean±SD of raw lactate concentrations. B: Lactate values over time with fitted regression lines; shaded areas represent the 95% confidence interval. Data are shown on the original (untransformed) scale for interpretability. Statistical inference was based on a linear regression model using log-transformed lactate values to satisfy model assumptions. *Indicates statistical significance (*p*<0.05).

**Figure 4.**
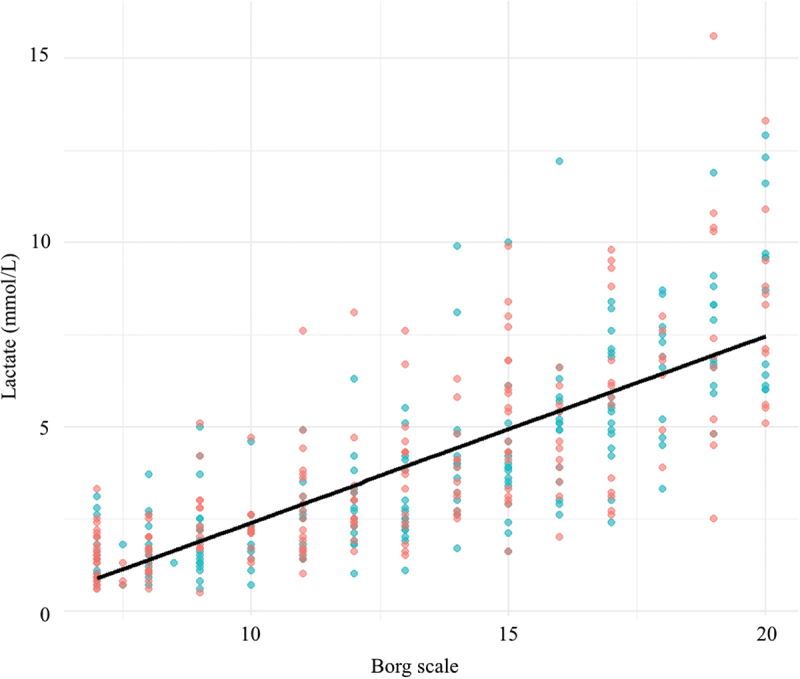
Correlation between Borg scale ratings and lactate levels.

Finally, we identified a strong positive correlation between Borg scale ratings and lactate levels (Rho: 0.77; *p* < 0.001). The findings suggest that higher lactate concentrations are associated with greater perceived exertion, reinforcing the physiological connection between these two variables during exercise (Figure 4).

Scatter plot showing the relationship between lactate levels and Borg scale ratings of perceived exertion during exercise. The black line represents the linear fit, emphasizing the trend of increasing lactate levels with higher Borg scale values.

## 4. Discussion

The main finding of our randomized controlled study involving seventy-one elite basketball players is that consuming a single dose of isotonic drink before exercise influences metabolic responses and subjective exertion without altering maximal cardiorespiratory parameters. As with most nutritional interventions, complete blinding was not feasible; therefore, some influence on subjective outcomes cannot be ruled out. Athletes consuming isotonic drinks reported lower perceived effort and demonstrated modestly reduced lactate accumulation compared to fasting conditions. Additionally, fasting progressively influences lactate accumulation during exercise, and there is a strong correlation between higher lactate levels and increased perceived exertion.

Athletic performance is shaped by a wide range of factors, including not only nutrition and hydration but also sleep, circadian rhythms, and cognitive load [28], which supports the ongoing investigation of nutritional interventions in athlete populations. To our knowledge, there is limited evidence on the effects of a single-dose isotonic drink consumed prior to short-term exercise in a controlled environment. Both the fasting and the isotonic drink group athletes demonstrated excellent cardiorespiratory fitness without any difference in maximal aerobic capacity. Most studies suggest that carbohydrate intake before and during exercise enhances performance; however, these studies examined the effects of the continuous use of isotonic drinks. Nicholas et al. demonstrated that consuming a 6.5% isotonic drink repeatedly during exercise, even after reaching fatigue, led to a 33% increase in running time [29]. Sports nutrition guidelines emphasize that the consistent consumption of isotonic drinks rather than a single dose may be necessary to achieve significant improvements in endurance and overall exercise performance [7,18]. In a similar placebo-controlled study with fewer athletes (eight men), carbohydrate-electrolyte solution with chromium picolinate improved performance and cognitive function, likely due to improved insulin sensitivity and glucose metabolism [30,31]. None of the above-mentioned studies examined the effect of a single-dose isotonic drink consumption prior to short-term exercise in a controlled environment. They also used different training protocols without the objective monitoring of physical demands.

The athletes in the isotonic drink group reported significantly lower exertion levels on the Borg scale, suggesting that pre-exercise carbohydrate consumption can reduce perceived exertion during high-intensity exercise. As with most nutritional interventions, complete blinding was not feasible; therefore, some influence on subjective outcomes cannot be ruled out. This limitation is particularly relevant when interpreting perceived exertion, as participants who consumed an isotonic drink may have expected improved performance, which could have biased their ratings. However, it is important to note that perceived exertion is not just a psychological construct, it is also closely linked to physiological stressors, such as lactate accumulation and metabolic fatigue [32]. In our study, Borg scores were significantly correlated with lactate levels, which supports the idea that the reported exertion reflects underlying metabolic processes. Although expectancy effects cannot be ruled out, the consistent association between subjective and objective markers indicates that Borg ratings remained physiologically relevant in this context. A study investigated the association between mouth rinsing with glucose and maltodextrin solutions, and exercise performance, and brain activity in cyclists [33]. The authors concluded that carbohydrate solutions may activate nerve receptors in the oral cavity, enhancing emotional and motivational feedback during exercise [33]. This mechanism may have contributed to our results; however, our athletes were not specifically asked to rinse with the drinks.

To monitor the metabolic status of the athletes, we measured the lactate levels not only pre-, peak-, and post-CPET but every 2 minutes during the exercise. While there was no difference in the pre-, peak- and post-CPET lactates, the 2-minute measurements showed that the fasting group experienced a larger magnitude of increase above the anaerobic threshold. This suggests that pre-exercise carbohydrate consumption may help to moderate lactate accumulation over time. Furthermore, our regression analysis revealed a significant connection between fasting and CPET duration, showing that fasting progressively increases lactate accumulation as exercising continues. As no influence was observed on peak lactate values, our results suggest that fasting may affect lactate kinetics rather than peak levels. As the average exercise time was 12.8 ± 1.6 minutes, the 14th-minute lactate levels represent athletes with the best performance in the group. Notably, while previous studies primarily demonstrated the benefits of pre-exercise carbohydrate intake during prolonged exercise, such as stabilized blood glucose levels and reduced reliance on anaerobic glycolysis [34,35], our findings raise the possibility that even a single dose of isotonic drink consumed before short, high-intensity exercise may influence lactate accumulation and help reduce perceived exertion. There is only one paper which investigated the effect of pre-exercise carbohydrate intake in 17 participants before a short-term cycling test and found an improvement in peak power output [36].

In our study, the lactate levels were measured alongside Borg scale ratings to examine how subjective perceptions of effort correspond with the objective markers of metabolic stress. We found a positive correlation between the Borg scale results and lactate levels, indicating that higher subjective exertion is associated with higher lactate concentrations, which is consistent with previous research results [37,38]. It is important to emphasize that lactate accumulation serves here as an indicator of anaerobic metabolism rather than as a direct cause of fatigue [13–15]. This connection underscores how physiological stress, as reflected by lactate levels, influences athletes’ subjective perception of effort during exercise [39]. This may be because carbohydrate intake in the form of isotonic drinks before exercise provides an immediate energy source, reducing the need to rely on muscle glycogen stores. Isotonic drink consumption thereby delays fatigue onset and likely accounts for the decreased perceived exertion and better lactate management, as seen in the isotonic drink group.

Our study also confirms that a small dose of carbohydrate intake before the CPET has no significant impact on the cardiorespiratory parameters. Although, the American Thoracic Society recommends two-hour fasting before the CPET, isotonic drink consumption or a small food intake might be safely used according to our results [11]. Moreover, existing literature shows that, on top of these factors, exercise modality may also significantly influence key physiological markers in athletes [40].

### 4.1. Strengths and limitations

Our study population includes elite male youth basketball players with similar training history and training schedule from one basketball academy. All players have organized training programs and diets supervised by the dietitian of the Academy. Due to this well-controlled organization our study population was homogenous without differences in baseline characteristics. The fasting and the isotonic groups were randomized equally regarding age.

Due to the controlled dietary program, all athletes received their primary meals at the Academy, which ensured consistency in carbohydrate intake between the groups. Our randomized controlled trial was supervised by the certified dietitian of the Academy, who is not only responsible for the dietary guidance of the team but also prepared the drinks for the players at the time of the exam.

To assess cardiorespiratory fitness, we used the most accurate exam, CPET complemented with pre-, peak-, and post-CPET glucose and lactate measurements. Lactate was also measured every two minutes along with the Borg scale recording to represent the metabolic status and the parallel perceived exertion during the test. This provided a reliable measure of performance under controlled laboratory conditions.

Regarding the limitations of the study, participants were not blinded to their group allocation, meaning that those in the fasting group were aware they were consuming mineral water. Ideally, a placebo beverage with a taste identical to the isotonic drink could have been used to enhance blinding. However, such a solution would require additives that might influence metabolic responses and thereby compromise the validity of the physiological measurements. Importantly, the physician conducting the CPET was blinded to group assignment, which helped minimize the risk of observer bias during test administration and evaluation. Blinding remains a recognized challenge in nutritional intervention studies, particularly in athletic populations [41].

## 5. Conclusions

In this study, we demonstrated that a single-dose of isotonic drink before CPET reduces the perceived exertion level and moderates lactate accumulation, suggesting beneficial effects on metabolic responses in youth athletes. Our results provide valuable insight into the nutritional needs and potential performance benefits of youth male basketball players, a population often underrepresented in sports nutrition research. These results indicate that even one isotonic drink before exercise testing may have beneficial effects; therefore, athletes should consider incorporating isotonic drinks into their pre-exercise hydration routine. Future well-controlled studies are needed to explore the long-term effects of isotonic drinks.

## Supplementary Material

Supplemental Material

## Data Availability

The dataset presented in this study are available on request from the corresponding author. Due to patient’s data, privacy data are not made available publicly.
