# Supplementary material for: Exercise with fasting or isotonic drink? A randomized controlled trial in youth elite basketball players
Source: J Int Soc Sports Nutr. 2025 Jul 8;22(1):2528533. doi: 10.1080/15502783.2025.2528533 (PMC12239237; doi:10.1080/15502783.2025.2528533)
Supplement: Supplemental Material [file RSSN_A_2528533_SM7150.docx]

Supplementary Figure 1. Q-Q plots assessing normality of CPET outcome variables.


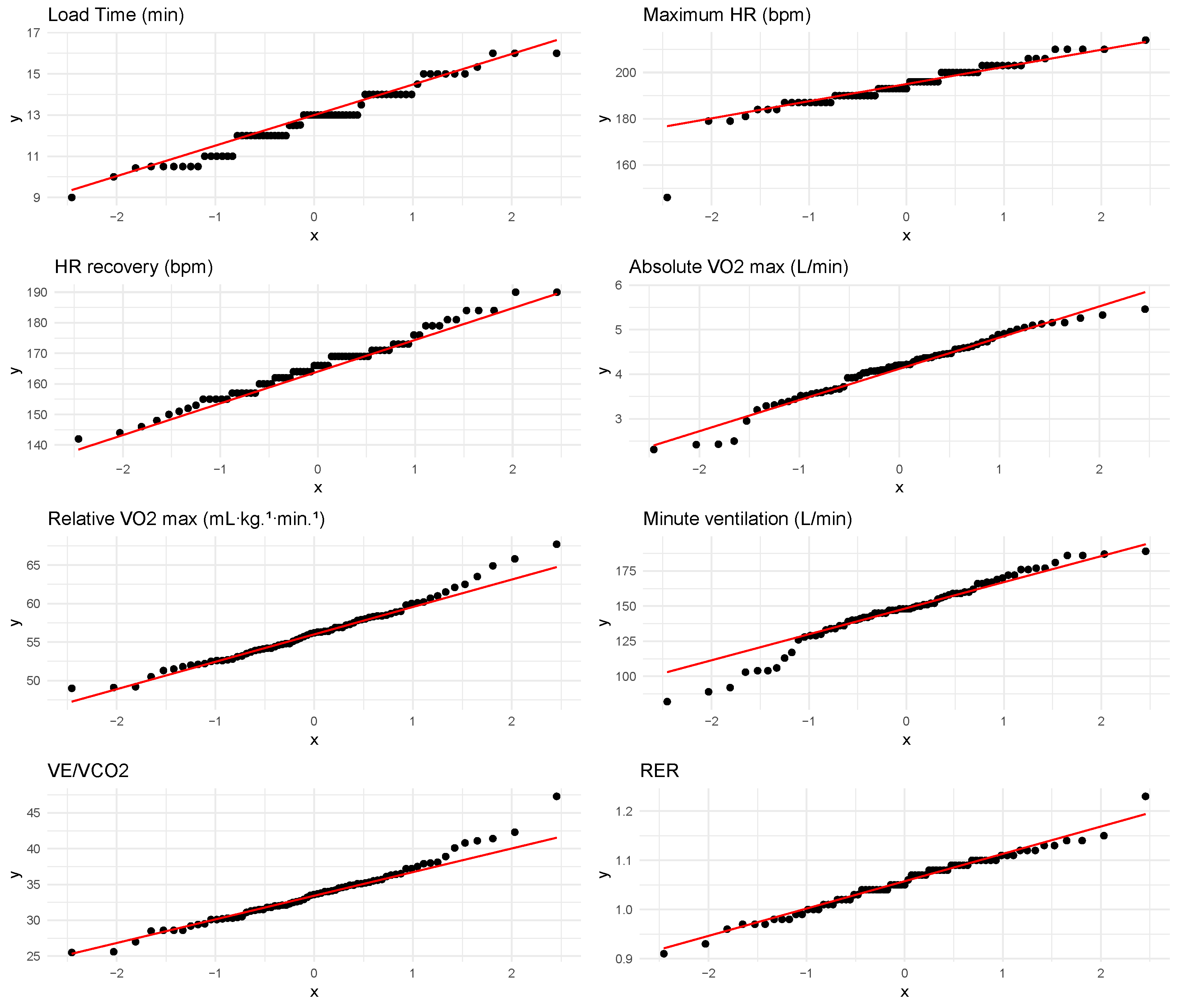


Supplementary Figure 2. Diagnostic plots for the linear regression model of Borg scale ratings.


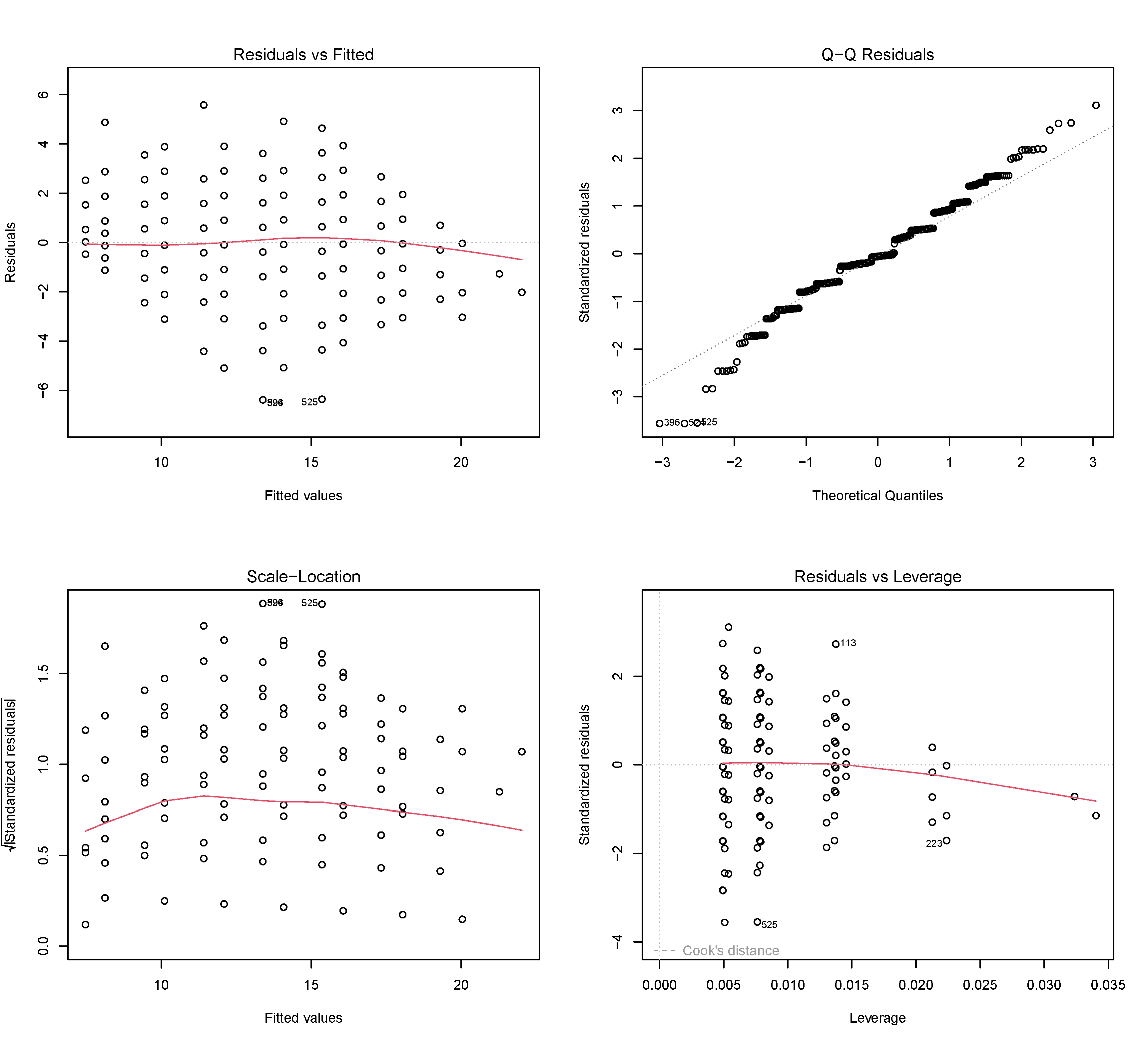


Footnote: Residual diagnostics for the linear model assessing the association between Borg scale scores and CPET load time by group. The plots indicate acceptable model fit, with approximately normal residuals, constant variance, and no evidence of influential outliers.

Supplementary Figure 3. Diagnostic plots for the linear regression model of log-transformed lactate values.


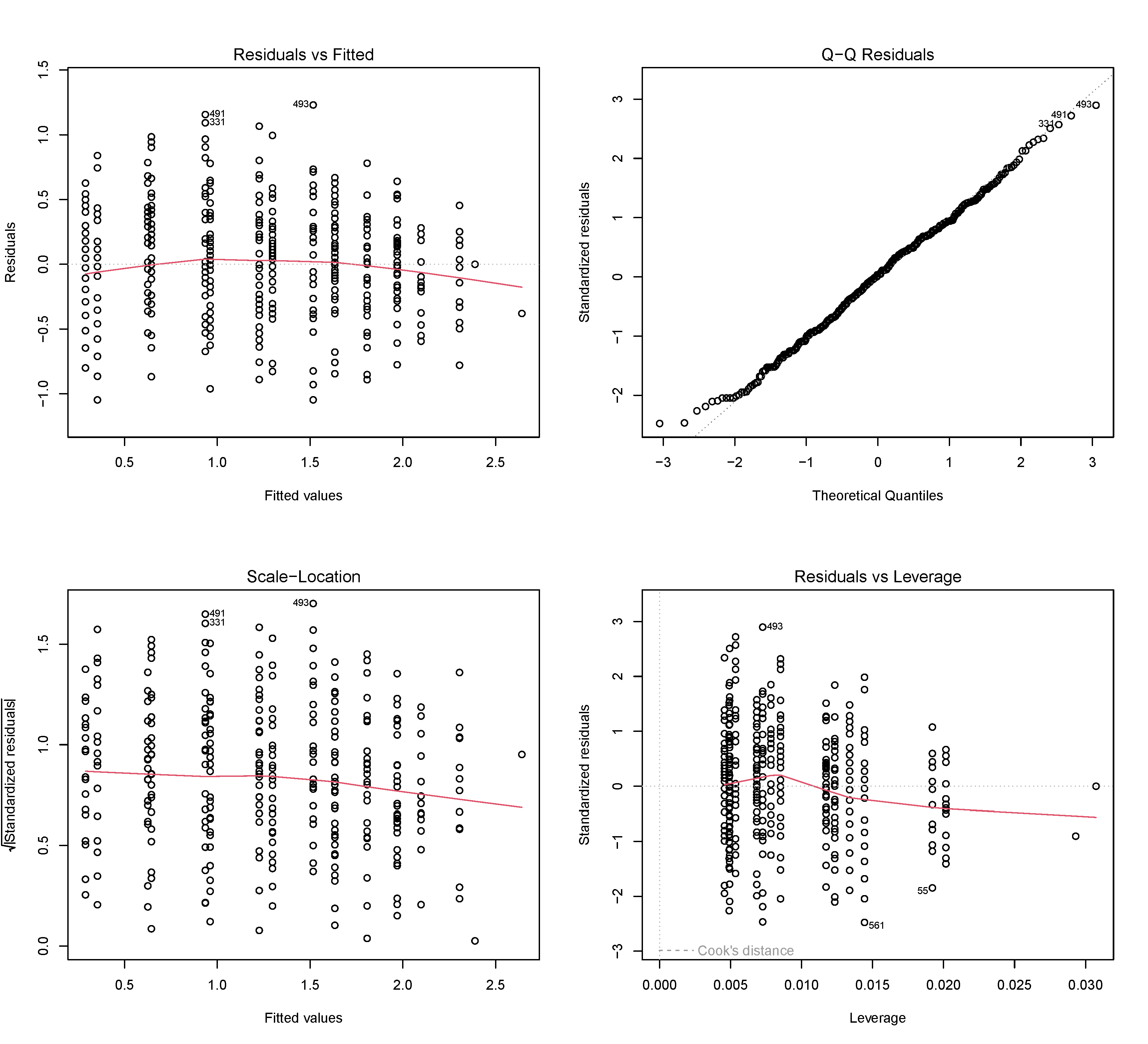


Footnote: Residual diagnostics for the model assessing the relationship between log-transformed lactate levels, load time, and fasting status. The Q-Q plot indicates normally distributed residuals, and the scale-location and residuals vs. leverage plots show no major heteroscedasticity or influential outliers, supporting the appropriateness of the linear model.

Supplementary Table 1. Association between drink group (isotonic vs. fasting) and cardiopulmonary exercise test (CPET) performance outcomes

| **CPET parameters** | **Coefficient** | **95% CI** | **Unadjusted**  **P value** | **FDR adjusted P value** |
| --- | --- | --- | --- | --- |
| Load Time | -0.26 | [-1.01, 0.49] | 0.490 | 0.843 |
| Maximum HR | -2.33 | [-6.97, 2.32] | 0.322 | 0.843 |
| HR recovery | 3.59 | [-1.33, 8.51] | 0.150 | 0.843 |
| Absolute VO2 max | -0.09 | [-0.42, 0.25] | 0.617 | 0.843 |
| Relative VO2 max | -0.82 | [-2.66, 1.02] | 0.375 | 0.843 |
| Minute ventilation | -1.43 | [-12.7, 9.85] | 0.802 | 0.911 |
| VE/VCO2 | 0.46 | [-1.44, 2.36] | 0.632 | 0.843 |
| RER | 0.00 | [-0.03, 0.03] | 0.911 | 0.911 |

Footnote: Linear regression models were used to assess the association between drink group (isotonic vs. fasting) and each CPET parameter. Coefficients represent the estimated mean difference between the isotonic and fasting groups. The table includes 95% confidence intervals (CI), unadjusted p-values, and false discovery rate (FDR)–adjusted p-values. No statistically significant differences were observed across groups. All outcome variables were confirmed to meet normality assumptions prior to analysis.
